# Editorial

**Published:** 1868-05

**Authors:** 


### Editorial.

---

MEDICAL SOCIETY MEETINGS.—The Indiana State Medical Society will meet at Indianapolis on Tuesday, May 19th, 1868.

The Illinois State Medical Society holds its next meeting in Quincy, commencing May 19th, 1868.

The Military Tract Medical Association holds its next meeting at Kewanee, on Tuesday, May 12th, 1868.

---

RICHMOND MEDICAL JOURNAL.—This excellent monthly medical periodical, an advertisement of which will be found in our advertising columns, can be furnished to the subscribers of the EXAMINER for $3.00 per annum.

Summer Teaching.—The Chicago Medical College has an excellent class of students attending the summer reading and clinical course of instruction.

---

Homœopathy and the Michigan University.—It is understood that the Board of Regents of Michigan University have finally accepted the appropriation of money by the Legislature of that State, which was made on the condition that a Chair of Homœopathy should be established as a part of the medical department of the University. This action has caused a part of the medical faculty to resign, and will probably break up the medical department entirely, or at least destroy all its importance. If it results in nullifying the medical department at Ann Arbor, and the establishment of an independent medical college in Detroit, where clinical instruction can be made a part of the regular course, it will be a positive benefit to the profession and to the people of that State.

If the remaining members of the present medical faculty in the University resign, it will be impossible for the Regents to fill their places in connection with their homœopathic projects; because neither the University nor its diploma will be longer recognized by other medical colleges and associations.

---

Medical College Graduates.—The following list shows the number of graduates, at the close of the last annual term of instruction, in most of the medical colleges in the United States:

| | |
|---|---|
| Jefferson Medical College, Philadelphia,--------- | 159 |
| Med. Department of University of Penn., Phila., | 153 |
| Rush Medical College, Chicago,------------------ | 116 |
| Bellevue Hospital Medical College, New York,-- | 111 |
| College of Physicians and Surgeons, " -- | 104 |
| Med. Dep't of University of Nashville, Tenn.,--- | 85 |
| Med. Department of New York University,----- | 82 |
| Med. Department of University of Michigan,---- | 76 |
| Medical College of Ohio, Cincinnati,------------ | 54 |
| Chicago Medical College,------------------------ | 50 |
| Massachusetts Medical College, Boston,--------- | 48 |

| | |
|---|---|
| Med. Dep't of University of La., New Orleans,- | 46 |
| St. Louis Medical College,---------------------- | 46 |
| Med. Dep't of University of Louisville, Ky.,---- | 46 |
| Buffalo Medical College, N.Y.,----------------- | 40 |
| Miami Medical College, Cincinnati,------------- | 29 |
| New Orleans School of Medicine,--------------- | 28 |
| Medical College of South Carolina,------------- | 27 |
| Charity Hospital Medical College, Cleveland,--- | 27 |
| Med. Dep't Harvard University, Boston,-------- | 26 |
| Missouri Medical College, St. Louis,------------ | 26 |
| Tolland Medical College, San Francisco,-------- | 13 |
| Cincinnati College of Medicine and Surgery,---- | 10 |

There are several other medical colleges in different parts of the country, not included in the above list. It is probable that the entire list would make the total number of regular medical college graduates in the United States, for the year 1868, about 1525.

---

Money Receipts to April 23d.—Drs. M. T. Darling, $6; W. H. Frazer, 3; Edmund Andrews, 3; W. H. Cook, 3; L. Humphreys, 3; F. L. Flanders, 3; W. P. Welsh, 3; S. H. Bottomly, 3; James Brewster, 6; Martin I. Whitman, 3; Orin Peak, 3; E. Dyson, 3; James Miner, 3; C. C. Crocker, 3; Mary H. Thompson, 3; H. H. Deming, 3.

### FLUID EXTRACTS.

The attention of physicians has been turned of late to the general unreliability, and, sometimes, entire worthlessness of the fluid extracts in common use. This inefficiency may be due to the quality of the drug used, or the dishonesty of the manufacturer, but is in most cases, more probably, the result of the mode of preparation. The trouble seems to lie with those drugs whose medicinal effect depends on volatile principles, which would be envolved on application of even a low degree of heat.

Here then is the difficulty that the use of heat renders the extract valueless, because it deprives it of its only valuable ingredient. To dispense then with this dangerous agent is the effort of every manufacturer.

Different makers have adopted different methods, involving the use, however, of more or less heat, but none have achieved the result desired, till Dr. Samuel P. Duffield, of Detroit, announced his process in which he avoids the use of any heat whatever. The following is a short description of this valuable improvement:

"The drug is ground to a coarse powder and placed dry in an iron cylinder. The air is then exhausted by means of an air pump, causing the pores of the drug to give up the air contained in them, and permit the entrance of the menstrum, which is forcibly sucked in through a syphon tube. The effect of this is to impregnate the menstrum with the entire soluble and medicinal properties of the drug, and thus rendering after concentration with the aid of heat unnecessary."

The theory of the above process seems clearly superior to others in use, and the practical workings of it, produce results as we would anticipate.

Many of our leading physicians, among whom we might mention Professors Weber and Scott, of Cleveland, Professor Armor, of Michigan University, Professor Hildreth, of Chicago, have tried fluid extracts made according to Dr. Duffield's process, by Duffield, Parke & Co., of Detroit, with a view to thoroughly test their merits, and have pronounced them decidedly superior to others in use.

In general appearance they differ much from the dark-colored preparations to which we are accustomed. The standard is that of the U. S. Pharmacopœa, sixteen troy ounces of the drug to the fluid pint.—*The Cincinnati Lancet and Observer.*

---

GONORRHŒA.—*Starch Injections.*—Finely powdered starch mixed with lukewarm water, so as to obtain a fluid of the substance of cream, but thin enough to allow of injection, forms a most successful injection in cases of gonorrhœa, especially after the inflammatory stage is over.—[M. Luc.]—*Braith. Ret.*

---

ECZEMA.—*Iodide of Lead.*—Iodide of lead is a remedy of great value in eczema. It should be applied in the form of an ointment, 12 grains to the ounce, with 1 drachm of glycerine, and 40 minims of chloroform, to relieve the itching. Another formula is the following: Iodide of lead, 20 grains; simple ointment, 7 drachms; glycerine, 1 drachm. The ointment of iodide of lead of the present pharmacopœia is too strong for cases of chronic eczema or psoriasis; it contains 62 grains to the ounce; whereas, from a-fifth to a-fourth of that quantity is sufficient, and more useful than the pharmacopœial strength. The use of constitutional treatment must be combined with this.—[Dr. W. T. Belcher.]—*Braithwaite's Retrospect.*

### ANNUAL MEETING OF THE BOARD OF HEALTH.

### ELECTION OF OFFICERS.

### REPORT OF THE HEALTH OFFICER FOR THE YEAR ENDING APRIL 1, 1868.

The Annual Meeting of the Board of Health was held on the 6th of April, 1868, Mayor Rice in the chair, and present, Commissioners Rauch, Wagner, Giles, Hoard, and Reynolds.

On motion of Commissioner Reynolds, the Mayor was unanimously reëlected the President of the Board.

The Committees on finance, ordinances, sanitary matters, and streets and alleys, were filled with the same members as on last year. They are as follows:—

*Sanitary Committee.*—Dr. Rauch, Dr. Johnson, Dr. Wagner.

*Finance Committee.*—Messrs Hoard, Giles, and Rauch.

*Committee on Ordinances.*—Messrs Johnson, Hoard, and Reynolds.

*Committee on Streets and Alleys.*—Messrs Giles, Reynolds, and Hoard.

On motion, Ambrose Burnham was reëlected health officer until April 1, 1869, and J. S. Kline was reëlected secretary of the Board for the same period.

Applications from S. W. Lee, G. W. Bonham, and E. Wood, for situations as sanitary policemen, were referred to the committee on appointments.

The following is the report of A. Burnham, health officer, for the year ending March 21, 1868:—

HEALTH OFFICE, CHICAGO, *April 1, 1868.*

*To the Honorable the Board of Health of the City of Chicago:*

GENTLEMEN,—I have the honor to present the following report for the last municipal year, of work done from the first day of April, 1867, to the first day of April, 1868:—

NUMBER OF NUISANCES REPORTED AND ABATED ON NOTICES.

| | | | |
|---|---|---|---|
| Ashes and rubbish on streets, yards and alleys, | 1,514 | Filthy houses, | 301 |
| Garbage, | 1,313 | Filthy hog-pens, | 65 |
| Manure piles, | 10,653 | Filthy hide stores and cellers, | 45 |
| Full privies, | 8,192 | Filthy privies, | 1,722 |
| Filthy alleys and areas | 4,079 | Filthy premises, | 47 |
| Filthy butcher shops, | 6 | Filthy packing houses, | 7 |
| Filthy basements, | 63 | Filthy soap factories, | 3 |
| Filthy cellars, | 63 | Filthy stables, | 1,117 |
| Filthy catch basins, | 6 | Filthy sinks, | 735 |
| Filthy cesspools and cisterns | 135 | Filthy slaughter-houses, | 5 |
| Filthy drains, | 534 | Filthy tanneries, | 5 |
| Filthy distilleries, | 6 | Filthy vacant lots, | 326 |
| Filthy gutters on streets & alleys, | 1,457 | Filthy water closets, | 348 |
| Filthy grocery stores, | 10 | Total, | 32,808 |

Number of notices served to connect premises with sewers, ---------- 1,859
Premises connected, -------- 1,097 | New privy vaults built, -------- 853
Total number of all notices served, ---------------------------------- 35,520
Loads of ashes removed, 7,247, about equally divided among all the divisions of the City:—

| | |
|---|---|
| Loads of ashes removed from the South Division, | 6,318 |
| Loads of ashes removed from the West Division, | 750 |
| Loads of ashes removed from the North Division, | 698 |
| Total loads of ashes removed, | 7,769 |
| Barrels of rotten apples, potatoes, eggs, fish, and pickles, | 1,342 |
| Baskets and boxes of decayed peaches, grapes, oranges, and lemons, | 773 |
| Barrels and bags of damaged poultry removed, | 36 |

Number of dead animals removed by City scavengers:—

| | | | | | |
|---|---|---|---|---|---|
| Horses, | 148 | Hogs, | 49 | Chickens, | 4090 |
| Sheep, | 67 | Dogs and cats, | 7093 | | |
| Cows, | 24 | | | Total, | 12,281 |

| | | | |
|---|---|---|---|
| Main sewers disinfected, | 3 | Sloughs and slips disinfected, | 35 |
| Private sewers disinfected, | 1735 | Total, | 1773 |
| Miles of streets and alleys disinfected, | 1146 | Houses disinfected, | 274 |
| | | Privies disinfected, | 2150 |

SMALL-POX.

The cases of small-pox in the City during the year were as follows:—

| | | | |
|---|---|---|---|
| 1st Ward | 27 | 10th Ward | 44 |
| 2d Ward | 112 | 11th Ward | 90 |
| 3d Ward | 97 | 12th Ward | 43 |
| 4th Ward | 58 | 13th Ward | 13 |
| 5th Ward | 153 | 14th Ward | 43 |
| 6th Ward | 104 | 15th Ward | 80 |
| 7th Ward | 170 | 16th Ward | 36 |
| 8th Ward | 74 | | |
| 9th Ward | 46 | Total, | 1193 |

The cases as reported by months were as follows:—

| | | | | | |
|---|---|---|---|---|---|
| April, | 28 | August, | 36 | December, | 157 |
| May, | 20 | September, | 23 | January, | 228 |
| June, | 39 | October, | 47 | February, | 247 |
| July, | 47 | November, | 137 | March, | 184 |

VARIOLOID.

The varioloid cases in the several wards were as follows:—

| | | | | | |
|---|---|---|---|---|---|
| 1st Ward | 22 | 7th Ward | 86 | 13th Ward | 2 |
| 2d Ward | 48 | 8th Ward | 48 | 14th Ward | 31 |
| 3d Ward | 56 | 9th Ward | 17 | 15th Ward | 29 |
| 4th Ward | 34 | 10th Ward | 21 | 16th Ward | 22 |
| 5th Ward | 35 | 11th Ward | 35 | | |
| 6th Ward | 62 | 12th Ward | 10 | Total, | 528 |

The total number of varioloid and small-pox cases in the City for the year was 1721.

The total number of patients removed to the small-pox hospital was 303.

The number of houses under sanitary inspection, where small-pox patients reside, were 1488.

The number of summons served for violation of the health ordinances was 1551.

The amount of fines assessed for the year was $5576,20.

A. BURNHAM, Health Officer.

MORTALITY REPORT FOR THE MONTH OF MARCH:—

CAUSES OF DEATH.

| Cause | Number |
|---|---|
| Accident, drowned, -- | 2 |
| Accident, fall ------- | 1 |
| Accident, fall by horse | 1 |
| Accident, gunshot.--- | 2 |
| Accident, suffocation - | 1 |
| Accident, C.B.&Q.RR. | 1 |
| Anæmia ------------ | 1 |
| Angina ----------- | 1 |
| Apoplexy ---------- | 6 |
| Atelectasis, pulmenium | 1 |
| Bowels, inflammation- | 5 |
| Brain, congestion of-- | 3 |
| Brain, disease of------ | 2 |
| Brain, effusion, ------ | 1 |
| Brain, inflammation - | 3 |
| Brain, softening of, -- | 1 |
| Births, still & prematu. | 45 |
| Bright's disease------ | 2 |
| Bronchitis ---------- | 7 |
| Bronchitis, capillary - | 1 |
| Bronchitis, vessecular, | 1 |
| Cancer ------------ | 1 |
| Cancer, of face------- | 1 |
| Cancer, of intestines-- | 1 |
| Cancer, of stomach --- | 2 |
| Cancer, of uterus----- | 3 |
| Cholera infantum ---- | 1 |
| Convulsions--------- | 42 |
| Convulsions, puerperal with sanguineous effusion ------------ | 1 |
| Croup-------------- | 9 |
| Croup, membranous-- | 2 |
| Debility ----------- | 5 |
| Delirium tremens ---- | 3 |
| Deficient vitality ---- | 1 |
| Diarrhœa ---------- | 4 |
| Diphtheria---------- | 4 |
| Dropsy ------------ | 2 |
| Dysentery, following Measles --------- | 1 |
| Dysentery ---------- | 1 |
| Encephalites -------- | 2 |
| Enteritis ---------- | 1 |
| Epilepsy ---------- | 2 |
| Erysipelas --------- | 1 |
| Exhaustion --------- | 1 |
| Exposure----------- | 1 |
| Endometritis -------- | 1 |
| Fever, intermittent -- | 1 |
| Fever, puerperal ---- | 3 |
| Fever, puerperal and brondirlis ------- | 1 |
| Fever, remittent----- | 1 |
| Fever, scarlet ------- | 7 |
| Fever, typhoid ------ | 10 |
| Fever, typhoid with spinal fistula ------ | 1 |
| Fever, typhus ------- | 2 |
| Fungus hemadotes --- | 1 |
| Gangrene----------- | 1 |
| Gastritis ----------- | 1 |
| Gastritis, chronic ---- | 1 |
| Gastrœntrites ------- | 1 |
| Hemorrhage, umbilical | 1 |
| Heart, disease of----- | 3 |
| Heart, fatty degenera. | 1 |
| Heart, organic disease | 1 |
| Heart, volvular disease | 2 |
| Hypertrophy of heart | 2 |
| Hemiplegia -------- | 1 |
| Hepatitis ---------- | 2 |
| Hydrocephalus ----- | 3 |
| Hydrocephalus, acute | 3 |
| Hydrocephalus, chronic | 1 |
| Inanition----------- | 1 |
| Indigestion -------- | 1 |
| Kidneys, disease of--- | 1 |
| Kidneys, inflammation | 1 |
| Laryngitis --------- | 1 |
| Laryngitis, chronic -- | 1 |
| Liver, atrophy of---- | 1 |
| Liver, abscess of----- | 1 |
| Liver, inflammation of | 1 |
| Lungs, congestion of - | 3 |
| Lungs, œdema of ---- | 1 |
| Lungs, paralysis of, following congestion of the brain --------- | 1 |
| Malformation ------ | 1 |
| Measles ----------- | 21 |
| Measles, following the | 1 |
| Meningitis---------- | 5 |
| Meningitis, cerebro-spinal ----------- | 4 |
| Meningitis, tuberculous | 4 |
| Nephritis ---------- | 2 |
| Œdema glottitis ----- | 1 |
| Old Age ----------- | 8 |
| Paralysis ---------- | 2 |
| Peritonitis --------- | 5 |
| Pericarditis -------- | 1 |
| Phthisis pulmonalisis- | 42 |
| Pleurisy ----------- | 2 |
| Pneumonia --------- | 20 |
| Pneumonia, broncho - | 1 |
| Pneumonia, pleuro--- | 2 |
| Pneumonia, typhoid-- | 2 |
| Pneumonia, following Measles --------- | 3 |
| Pyemia ----------- | 1 |
| Rheumatism ------- | 1 |
| Scrofula ---------- | 1 |
| Small-pox -------- | 35 |
| Suicide ----------- | 2 |
| Stomach, inflammation | 1 |
| Tabes mesenterica --- | 4 |
| Teething ---------- | 1 |
| Uræmia ----------- | 1 |
| Varioloid---------- | 2 |
| Whooping-cough ---- | 1 |
| Total --------- | 379 |

Deaths in March 1868, --379 | Deaths in March 1867, --280 | Increase, -- 99

Deaths in February, 1868,------ 423 | Decrease, -------------------- 44

AGES.

| Age | Number |
|---|---|
| Under 5 ----------- | 190 |
| 5 to 10 ----------- | 31 |
| 10 to 20 ---------- | 16 |
| 20 to 30 ---------- | 39 |
| 30 to 40 ---------- | 38 |
| 40 to 50 ---------- | 22 |
| 50 to 60 ---------- | 18 |
| 60 to 70 ---------- | 10 |
| 70 to 80 ---------- | 13 |
| 80 to 90 ---------- | 2 |
| 90 to 100 --------- | 0 |
| 100 to 110 -------- | 0 |
| Unknown --------- | 0 |
| Total --------- | 379 |

Males,------------203 | Females,----------176 | Total,-----------379

Single,-----------278 | Married,--------- 101 | Total,------------379

White,-----------373 | Colored,----------- 6 | Total,------------379

NATIVITY.

| | | | | | |
|---|---|---|---|---|---|
| Chicago | 175 | Holland | 3 | Prussia | 4 |
| Other parts U. S. | 73 | Ireland | 37 | Scotland | 2 |
| Bohemia | 1 | Island of Jamaica | 1 | Sweden | 1 |
| Canada | 4 | Norway | 7 | Unknown | 2 |
| England | 14 | New Brunswick | 1 | | |
| Germany | 54 | | | Total | 379 |

DEATHS BY SMALL-POX.

For the Month of March, 1868.

| | | | |
|---|---|---|---|
| 2d Ward | 2 | 12th Ward | 2 |
| 3d Ward | 3 | 14th Ward | 1 |
| 4th Ward | 1 | 15th Ward | 3 |
| 5th Ward | 8 | Lake Hospital | 7 |
| 7th Ward | 4 | Unknown | 1 |
| 8th Ward | 1 | | |
| 11th Ward | 2 | Total, | 35 |

Varioloid, 14 Ward, 2

MORTALITY BY WARDS FOR THE MONTH.

| Ward. | Mortality. | Pop. in 1866. | One death in | Ward. | Mortality. | Pop. in 1866. | One death in |
|---|---|---|---|---|---|---|---|
| 1 | 7 | 9,668 | 1,381 3-7 | 14 | 17 | 12,108 | 712 1-4 |
| 2 | 15 | 12,985 | 865 2-3 | 15 | 38 | 15,766 | 414 9-10 |
| 3 | 24 | 15,738 | 655 3-4 | 16 | 33 | 14,912 | 451 7-8 |
| 4 | 30 | 10,884 | 362 4-5 | County hosp. | 5 | | |
| 5 | 24 | 9,610 | 400 5-12 | HosWom.Chi. | 0 | | |
| 6 | 24 | 10,680 | 440 4-5 | Soldier's Ho. | 1 | | |
| 7 | 30 | 18,755 | 625 1-6 | Home of the Friendl's, | 0 | | |
| 8 | 23 | 10,429 | 453 10-23 | | | | |
| 9 | 12 | 13,940 | 1 161 2-3 | Marine hos., | 1 | | |
| 10 | 17 | 11,416 | 671 1-2 | Mercy hos., | 5 | | |
| 11 | 30 | 12,924 | 430 4-5 | St. Luke's hos. | 0 | | |
| 12 | 18 | 12,695 | 705 1-4 | St. Jose. asyl. | 3 | | |
| 13 | 14 | 8,188 | 584 6-7 | Lake Hosp., | 7 | | |

Total, 379

The Report was ordered placed on file.

Commissioner Reynolds, from the committee on appointments, recommended the appointment, as sanitary police inspectors, of H. N. Alexander and James E. Newland. The report was adopted, and the appointments made.

The Board then adjourned until 10 o'clock on Monday, when the report of expenditures and the annual mortality report will be rendered.

---

ILLINOIS STATE MEDICAL SOCIETY.—The next Annual Meeting of the Illinois State Medical Society will be held in the City of *Quincy*, commencing on the *Third Tuesday* in May, 1868. It is very desirable that the profession throughout the Stateshould be well rep resented.

N. S. DAVIS,

*March 26th, 1868.* *Permanent Sec'y.*

INFLUENCE OF ALPINE CLIMATES ON PULMONARY CONSUMPTION.—Some startling cases are related, showing the beneficial influence of Alpine climates on pulmonary consumption. One patient lost his cough and gained flesh on the Peruvian Andes, and, after a subsequent relapse at New Orleans, entirely recovered his health on the table-land of Mexico. A second patient, after spending a winter at Bordeaux and Cannes without entirely losing his cough, and where he still continued to lose weight, went to the Righi, in Switzerland, where he lived almost entirely in the open air and drank about two quarts of milk a-day. He recovered his health perfectly, so far as his own sensations went. The third case went to Davos am Platz, and recovered. The treatment adopted there by Drs. Spengler and Unger, is the use of much milk and light nourishing food; a moderate amount of wine, principally the red wine of the Valteline; and graduated exercise, first on level, later up hill. The cold douche is likewise, in many cases, used with advantage. It is probable that in addition to the exhilaration and increased inclination to exercise produced by the cooling, refreshing atmosphere and the complete change in the manner of living, that the increased exercise of the respiratory organs, the fuller, deeper inspirations necessitated by the rarity of the atmosphere influences the respiratory circulation and all the processes of nutrition.—[Dr. H. Weber.]—*Braithwaite's Ret.*

